# Technologies to Support Community-Dwelling Persons With Dementia: A Position Paper on Issues Regarding Development, Usability, Effectiveness and Cost-Effectiveness, Deployment, and Ethics

**DOI:** 10.2196/rehab.6376

**Published:** 2017-01-16

**Authors:** Franka Meiland, Anthea Innes, Gail Mountain, Louise Robinson, Henriëtte van der Roest, J Antonio García-Casal, Dianne Gove, Jochen René Thyrian, Shirley Evans, Rose-Marie Dröes, Fiona Kelly, Alexander Kurz, Dympna Casey, Dorota Szcześniak, Tom Dening, Michael P Craven, Marijke Span, Heike Felzmann, Magda Tsolaki, Manuel Franco-Martin

**Affiliations:** ^1^ Department of Psychiatry VU University medical centre Amsterdam Netherlands; ^2^ Universities of Salford and Stirling UK Manchester, Stirling United Kingdom; ^3^ School of Health and Related Research (ScHARR) University of Sheffield Sheffield United Kingdom; ^4^ Institute for Ageing Newcastle University Newcastle United Kingdom; ^5^ Department of General Practice and Elderly Care Medicine VU university medical centre Amsterdam Netherlands; ^6^ Iberian Research Psychosciences Institute Psychosocial Rehabilitation Centre, Intras Foundation Zamora Spain; ^7^ Alzheimer Europe Luxembourg Luxembourg; ^8^ German Center for Neurodegenerative Diseases (DZNE), Site Rostock Greifswald Germany; ^9^ Association for Dementia Studies University of Worcester Worcester United Kingdom; ^10^ Centre for Person-centred Practice Research Queen Margaret University Edinburgh United Kingdom; ^11^ Technische Universität München Munchen Germany; ^12^ School of Nursing and Midwifery National University of Ireland Galway Galway Ireland; ^13^ Department of Psychiatry Wroclaw Medical University Wroclaw Poland; ^14^ Division of Psychiatry & Applied Psychology University of Nottingham Nottingham United Kingdom; ^15^ NIHR MindTech Healthcare Technology Co-operative Institute of Mental Health University of Nottingham Innovation Park Nottingham United Kingdom; ^16^ Bioengineering Research Group Faculty of Engineering University of Nottingham Nottingham United Kingdom; ^17^ Windesheim University of Applied Sciences Zwolle Netherlands; ^18^ National University of Ireland Galway Galway Ireland; ^19^ Memory and dementia outpatient clinic 3rd Department of Neurology Aristotle University of Thessaloniki Thessaloniki Greece; ^20^ Iberian Research Psychosciences Institute Psychiatric Department in Zamora Hospital Salamanca University Zamora Spain

**Keywords:** dementia, technology, evaluation studies, diffusion of innovation, ethics

## Abstract

**Background:**

With the expected increase in the numbers of persons with dementia, providing timely, adequate, and affordable care and support is challenging. Assistive and health technologies may be a valuable contribution in dementia care, but new challenges may emerge.

**Objective:**

The aim of our study was to review the state of the art of technologies for persons with dementia regarding issues on development, usability, effectiveness and cost-effectiveness, deployment, and ethics in 3 fields of application of technologies: (1) support with managing everyday life, (2) support with participating in pleasurable and meaningful activities, and (3) support with dementia health and social care provision. The study also aimed to identify gaps in the evidence and challenges for future research.

**Methods:**

Reviews of literature and expert opinions were used in our study. Literature searches were conducted on usability, effectiveness and cost-effectiveness, and ethics using PubMed, Embase, CINAHL, and PsycINFO databases with no time limit. Selection criteria in our selected technology fields were reviews in English for community-dwelling persons with dementia. Regarding deployment issues, searches were done in Health Technology Assessment databases.

**Results:**

According to our results, persons with dementia want to be included in the development of technologies; there is little research on the usability of assistive technologies; various benefits are reported but are mainly based on low-quality studies; barriers to deployment of technologies in dementia care were identified, and ethical issues were raised by researchers but often not studied. Many challenges remain such as including the target group more often in development, performing more high-quality studies on usability and effectiveness and cost-effectiveness, creating and having access to high-quality datasets on existing technologies to enable adequate deployment of technologies in dementia care, and ensuring that ethical issues are considered an important topic for researchers to include in their evaluation of assistive technologies.

**Conclusions:**

Based on these findings, various actions are recommended for development, usability, effectiveness and cost-effectiveness, deployment, and ethics of assistive and health technologies across Europe. These include avoiding replication of technology development that is unhelpful or ineffective and focusing on how technologies succeed in addressing individual needs of persons with dementia. Furthermore, it is suggested to include these recommendations in national and international calls for funding and assistive technology research programs. Finally, practitioners, policy makers, care insurers, and care providers should work together with technology enterprises and researchers to prepare strategies for the implementation of assistive technologies in different care settings. This may help future generations of persons with dementia to utilize available and affordable technologies and, ultimately, to benefit from them.

## Introduction

Due to our aging societies, dementia has become a 21st-century global public health concern, placing considerable burden on not only the individual and their family but also current and future service provision [1]. Worldwide prevalence is around 46 million, a figure predicted to treble to 131.5 million by 2050, with current care costs recently estimated at US $818 billion [2]. Among all chronic diseases, dementia is one of the most important contributors to dependence, disability, and care home placement [3]. Despite a global policy push toward more timely diagnosis and earlier intervention, considerable geographical inequalities in the provision of post-diagnostic care and support services exist [4]. One aspect of postdiagnostic support, which may enable persons with dementia to remain independent for a longer time and thus potentially leading to cost savings by delaying entry into care and nursing homes [2,3], is assistive technology. Assistive technology for persons with dementia can be defined as “Any item, piece of equipment, product or system driven by electronics, whether acquired commercially, off-the-shelf, modified or customized, that is used to help persons with dementia in dealing with the consequences of dementia” (see also Marshall [5]; Assistive Technology Industry Association [6]; ISO9999 [7]). The technology does not necessarily need to be “purposely designed” [8] for persons with dementia, as many mainstream technologies can be adapted to their changing needs. Important need areas in dementia are memory support, information, company, reducing psychological distress, and engaging in daytime activities [9,10]. Various technologies have been developed to address these needs, such as electronic calendars, Web-based information systems, video-calling, and electronic activity support systems [11-13].

Evaluation studies have found that persons with dementia are positive about using electronic devices to facilitate their independence and reduce family stress [11,14]. Furthermore, small-scale studies have found that assistive technologies improve independence [15], behavioral symptoms in persons with dementia [16], and quality of life [15], and stress in carers [16].

Despite the promising benefits of technological support systems, several issues remain before they will really make a difference in the field of dementia care. For example, the predominant use of technological solutions for safety and security and carer reassurance rather than for lifestyle in general [17]; the slow uptake and implementation of assistive technologies; the lack of high-quality scientific research into the effectiveness and cost-effectiveness of assistive technologies in dementia care [18,19], the lack of successful commercialization of prototype technologies; and the limited attention to aesthetics, which can make many technological support systems feel stigmatizing [20]. Furthermore, professionals and society also seem to lack an applied understanding of the potential of assistive technology in dementia because it is not being integrated into mainstream dementia care practice [20,21].

The need to address these issues has been widely acknowledged. For example, joint research efforts on assistive technologies in dementia were identified via a taskforce on Assistive Technology setup within INTERDEM (an interdisciplinary European research network of more than 160 members, collaborating to develop and carry out pan-European research on early, timely, and quality psychosocial interventions in dementia [22]). Experts from this taskforce worked together to discuss and reach consensus regarding the current state of affairs regarding (assistive) technologies for community-dwelling people with dementia. This resulted in this position paper.

Based on literature and expert opinions, key areas were considered including development issues, usability, effectiveness and cost-effectiveness, deployment, and ethics of (assistive) technologies for community-dwelling people with dementia. The term “assistive technology” included a wide range of aids, appliances, and whole-system applications; consequently, discussions were focused on technologies that addressed the following 3 areas of global need:

1. Devices intended to help persons living with dementia to manage their everyday life across the disease journey, such as electronic calendars and reminders for activities, medication reminders, aids to perform activities of daily life, robots, and navigation systems.

2. Technologies to help people engage in meaningful and pleasurable activities such as cognitive stimulation and physical activities, as well as technologies to improve social participation, contact, and support.

3. Health care technologies that aim to support professional organizations and systems within dementia health and social care, such as behavior monitoring, shared decision making, and Global Positioning System (GPS) tracking systems.

We concluded with a set of recommendations for key stakeholders including the research community, technology developers (industry and business), care commissioners, and care providers to better prepare them to ensure the ongoing delivery of high-quality, efficient care and support to the growing numbers of persons living with dementia and their families.

## Methods

Literature reviews were performed by members of the taskforce Assistive Technology, who met twice (Ljubljana, September 2015; Berlin, October 2015) to discuss the aim and methodology of this study and divide the work. Each subsequent section was led by 2 taskforce members and prepared by a subgroup of the taskforce Assistive Technology.

The section on technology development was based on expert opinion and relevant literature, among other previous reviews of taskforce members [23,24]. For the sections on usability, effectiveness and cost-effectiveness, and ethical issues, separate literature searches were conducted in PubMed, CINAHL, PsycINFO, and Embase databases. Common search terms were used for dementia (“Dementia”[Mesh]) OR (dement* OR alzheimer* OR lewy OR CJD OR JCD OR creutzfeldt OR binswanger OR korsakoff OR frontotemporal OR FTD OR “vascular dementia” OR VaD OR “pick disease” OR “picks disease”) and technology ((assistiv* OR orthotic* OR supportiv* OR electronic*) AND (technolog* OR device*)) OR telecare OR “Self-Help Devices”[Mesh] OR (“information communication technology” OR ICT), added with specific terms for the sections on usability ((usability AND (computer OR technology OR software OR virtual reality)) and ethics (ethic*). Inclusion criteria were reviews in English, reporting (partly) on persons with dementia living in the community, and technologies in 1 or more of the 3 selected areas (daily living, meaningful and pleasurable activities, and health care technology). There was no restriction on publication dates, and the searches were finalized in January 2016.

All records from the searches were reviewed by at least two independent researchers in each section to check whether they should be included. Another researcher was involved to reach consensus in cases of disagreement. Reviews that met the inclusion criteria were included, and the results of the reviews (or single studies in the reviews if relevant) were summarized. For the section on deployment, searches were conducted in specific Health Technology Assessment databases, using the search terms: assistive technology dementia.

## Results

### Development Issues Regarding Assistive Technologies for Daily Living, Meaningful and Pleasurable Activities, and Health Care Technology

In the past, devices for older people were generally created by technologists with little attention to the specific needs of older end users, and thus the users’ requirements of devices. Nowadays, there is wider understanding of the importance of engaging end users at all stages of technology development to ensure their needs are addressed and to promote acceptance of technological aids. However, challenges in the development of technological devices were identified as follows: How can technologies address the heterogeneous needs of persons with dementia? Should technologies be designed specifically for dementia or adapted from mainstream technology? What methods are more efficacious when developing technologies for persons living with dementia? Finally, we addressed what challenges are to be faced regarding developmental issues in the 3 selected application areas of assistive technology.

#### Technologies to Address the Heterogeneous Needs of Persons With Dementia

To develop technologies that are useful and valuable for persons with dementia, it is important to know what kind of assistance is needed. This requires a thorough understanding of the different types of dementia and associated impairments, individual experiences and coping mechanisms, and the continuous changing situation during the dementia “journey.” It is also important to be attentive to needs such as a sense of self-esteem and feeling respected, which are related to higher levels of well-being and quality of life, as highlighted in Maslow’s “hierarchy of needs” [25,17]. People with dementia can express their needs [26] and preferences [27] consistently, even in an advanced stage of dementia [28]. Therefore, to really understand what it is to live with dementia and which needs should be addressed, people with dementia should be asked about their needs and experiences and be involved early in the process of development of supportive tools and interventions. Till now, very few technologies have actually been designed to meet the specific needs of people living with dementia [29], and only few of these prototypes have been adopted for commercial development.

#### Technologies Designed Purposively for Dementia or Adapted From Mainstream Technology

Technologies can be divided into those designed specifically for persons with dementia as opposed to technologies that have been developed in the mainstream and lend themselves well to support people with cognitive difficulties. For example, the functionality of some forms of telecare technology, such as GPS, webcams, and apps (Joint Improvement Team, 2016), is being superseded by readily accessible off-the-shelf devices that can successfully assist people to navigate their day. Also, recent work has confirmed that persons with dementia can be supported to use touchscreen computing for leisure and recreation in line with the rest of society [29]. Nevertheless, the complex sensory, perceptual, and cognitive changes caused by dementia can make using mainstream devices problematic for some persons with dementia, and therefore for the foreseeable future, some demand for bespoke devices will continue.

#### Methods of Technology Development in Dementia

In developing assistive technologies in health care, there has been a shift from expert- and technology-led design toward a user-driven approach, and it is more common to now involve end users.

Examples of methods that support end user involvement and aim for sustainable eHealth innovations are the holistic approaches of the roadmap of the Centre for eHealth Research and Disease Management (CeHRes) [30] and Contextual Design [31]. Both methods are rooted in human-centered design (HCD) and emphasize 3 interrelated components: technology, people, and organization (health care environment). The CeHRes roadmap focuses, in particular, on the health domain and combines HCD principles with business modeling.

For dementia, the drive to ensure engagement at all stages of technology development is underpinned by the principles of person-centered care and, in a broader perspective, by a social inclusive society. This includes the coproduction of new innovations for research and for practice, with the involvement of end users from the outset [32,33]. In practice, however, people with dementia have rarely been involved in technology development, with user acceptability tending to be assessed via family carers and others [11,24,34]. Successful examples of collaborative working with people with mild-to-moderate dementia are emerging [14,33,35-37]. However, people with more severe dementia are less often included in development of assistive technologies.

#### Challenges in the Development of Assistive Technology

Challenges in the development of assistive technology include the need for personalized and tailored technologies in dementia. A “one size fits all” is not an optimal solution because of the individual variations in needs and abilities. The development of sustainable assistive technology for persons with more severe dementia is a challenge, as is how to develop technologies in a way that will help to make the world a more “dementia-friendly” place [38]. Examples of assistive technologies that can help persons with dementia in their daily life are simple aids such as calendars and reminders but also more complicated devices such as robots that perform a social role or augment individual human capabilities through cognitive prosthetics [39]. There are companies who anticipate providing inclusive assistive technology solutions for older people, including those with dementia, for example, Alcove [40]. One research challenge is how to develop assistive technologies that address the emotional state of persons with dementia during everyday tasks [41]. One of the challenges in the field of health care technology, which supports organizational and supportive systems of dementia care, is to integrate technology into the built environment, such as lighting, floor coverings, and improved way-finding (eg, via improved signage), taking into account the varying and changing needs of the residents [42,43]. Another challenge is to integrate technology into the routine health care, using information and communication technology (ICT) in the clinical assessment of cognitive, behavioral, and physical functioning of persons with dementia [44].

#### Conclusion on State of Affairs Development of (Assistive) Technologies in Dementia

Research has revealed that persons with dementia are enthusiastic about using assistive technology to remain independent and also about taking part in technology design [23,33]. At the same time, some challenges remain, such as how to personalize and tailor technologies to the individual and changing needs and abilities of persons with dementia. We envisage that the involvement of end users in developing new assistive technologies will continue to grow, and that more applications of existing technology using mobile phones or apps will be put to use to benefit persons with dementia.

### Usability of Assistive and Health Technology in Dementia

**Figure 1:**
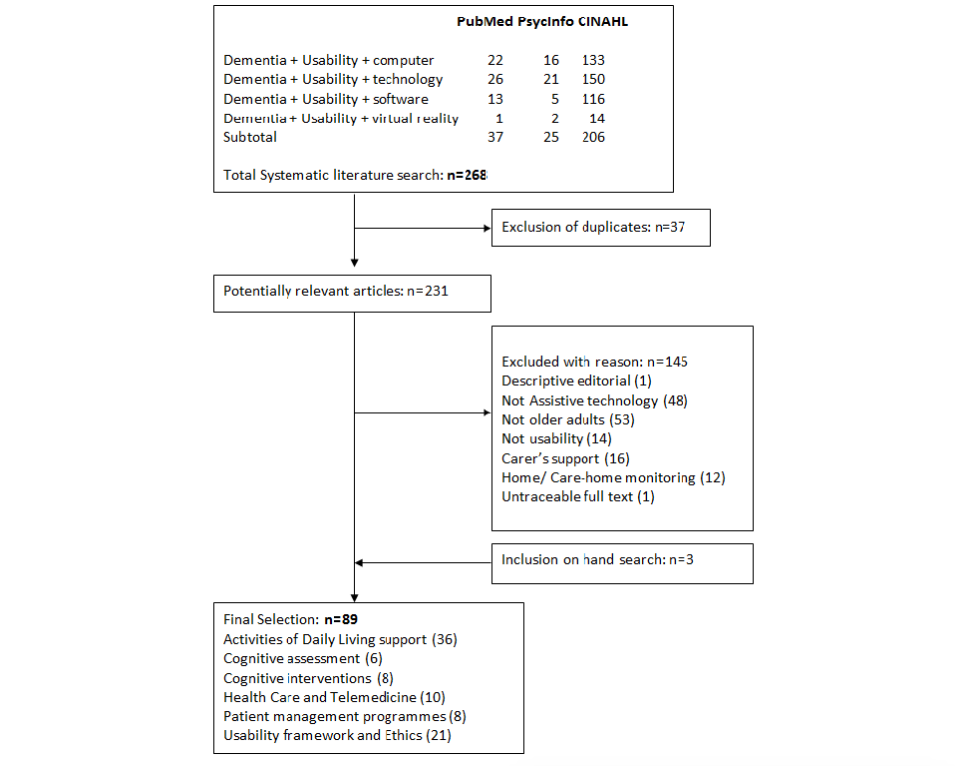
Flowchart of systematic review on usability.

The International Organization for Standardization defines usability as “the extent to which a product can be used by specified users to achieve specified goals with effectiveness, efficiency and satisfaction in a specified context of use” [45]. Thus, usability refers to the capability of the technology to be understood, learned, and used under specified conditions. The literature review on usability issues in dementia resulted in 89 papers (Figure 1). The main results are discussed in the following sections.

#### Usability of Technologies to Support Persons With Dementia in Everyday Life

Little research so far has been conducted in the field of assistive technologies in community dementia care and support, with only 3 studies exploring usability in supporting everyday life with a Day Navigator [46], a GPS [47] and a timer device [48]. In the study by Meiland et al, 42 participants and carers considered the Day Navigator to be mainly user-friendly, but conclusions about usefulness were limited due to insufficient duration of the testing period [46]. The GPS system was tested among 33 dyads, with only 1 leaving the study because of technical reasons. Participants with dementia who went outside unaccompanied took the GPS with them 67% of the time. Also, 80% (20/25) of the caregivers said that the use of the technology was not difficult, and almost all of them felt that they were in control of the secured website where they could track and trace their relatives (92%; 23/25). The study does not provide specific information about usability outcomes apart from ease and frequency of use and the fact that the participants with dementia did not seem to mind that they heard a voice from their GPS without notification [47]. The timer device was used for a stove and tested with 9 older adults with cognitive impairment or dementia and 5 relatives. The authors found that end users scarcely participated in the process of choosing and adapting the device. Although the device provided increased safety, there were also some unforeseen problems, such as not fully understanding how the device worked. The authors stressed the importance of actively involving users in home modifications with assistive technologies and providing medium- and longer-term follow-up of the technological support [48].

#### Usability of Technologies to Support Participation in Meaningful and Pleasurable Activities

In research on technology to participate in meaningful and pleasurable activities, for example, cognitive interventions for persons with dementia, usability issues are often not mentioned. Jelcic et al [49] reported a positive perception of technology-based cognitive therapy, as participants would recommend it to others and were satisfied with the utility and appeal of this intervention. Zaccarelli et al [50] found that the educational level of users was important, as results of the studies on people with Alzheimer’s disease, mild cognitive impairment and healthy adults showed that participants with a higher education level found it easier to learn how to use the ICT platform. Lee et al [51] reported that the usability of their computer-based cognitive intervention was good. Persons with dementia were highly motivated in using it, and their sense of achievement was enhanced; they took pride in showing others that they could operate the computer [51]. Gillespie et al [52] suggested that large-scale studies of assistive technology to improve cognition should also focus on functional areas, for example, prompting, navigating, and reminding, rather than on the specific content of the devices itself.

#### Factors Influencing Usability

Over time, persons with dementia may have reduced ability for new learning, which may impact actual use of technology because learning and technology use are inseparable and proceed together [53]. Understanding how persons with dementia access and embrace technology is vital in order to develop usable and acceptable technological solutions. Technology use by older adults has been criticized for not eliciting and including their interests [54]. Devices should be adjusted to each individual, achieving better tailored interventions, and assistive technologies should be embedded in a person-centred model [55]. A good example of this is the provision of feedback sessions to ensure that the person with dementia and carer understand the assistive technologies, to answer questions, and to collaboratively discuss recommendations for improvement [56]. A recent review (not limited to dementia) on mHealth applications suggested the adoption of automated evaluation mechanisms to improve the empirical methods to assess usability [57].

Furthermore, a good match between the person and the technology is required because if this is not achieved well from the end user’s perspective, the technology may be ignored or not be used optimally [58]. Bardram et al [59] emphasized the importance of deploying assistive technologies in a real-world setting, outside the laboratory, and also the need to perform longitudinal studies that assess the evolution of the relationship between the end user and the technology [59]. A person’s acceptance of assistive technologies can vary during the course of dementia. For example, acceptance can improve when symptoms start to threaten the independence of the person [60]. The ability to use assistive technology may also vary. Over time, a decreasing use of technology is seen in people with cognitive impairment [61].

It has been suggested that usability studies of assistive technologies should be designed in several stages: predeployment (observation sessions, focus groups with people with dementia, carers, and professionals); deployment (carrying out long-term observations and quantitative and qualitative assessments in real settings); and postdeployment (feedback sessions) in close partnership with end users, carers, and specialists [62].

#### Usability in the Area of Computer Technologies

In the area of computer technologies, usability of interfaces has received special attention. Research on the preferences of persons with dementia has indicated that touchscreen devices are preferred over mouse or keyboard input devices [63]. Direct response devices using a touchscreen reduce the distance between the subject (seeing the stimuli) and the causal effect (providing the answer), which enhances the person´s involvement in the task. The previous experience of people with dementia with computers affects which type of interface device they prefer, with experienced users preferring the mouse. However, although the mouse is the most demanding device in terms of cognitive and motor demands, there can also be problems with touchscreens in terms of accuracy that may be frustrating for the end user [64]. Computer literacy has an important role in usability: lack of computer experience was reported to decrease the odds of successful use of technology [65]. Thus, pretest, treatment, or intervention training sessions could be used to enable persons with mild cognitive impairments and early dementia to become familiar with novel technologies [66-69]. The need for including performance tests to enhance the ecological validity of assistive technologies has been highlighted, such as measuring the user’s motivation [54]. Although there is a prejudice that assistive technologies are not “elderly friendly,” in fact the evidence points in the opposite direction: when older adults get the opportunity to use computers, they regard them as a “status symbol” often associated with youth; as a consequence, the use of computers could have a positive effect on self-confidence and self-esteem [70].

Regarding the assessment of the usability of assistive technologies and user satisfaction, various tools were used, for example, the usefulness, satisfaction and ease of use questionnaire [71]; the Everyday Technology Use Questionnaire [72]; the Quebec User Evaluation of Satisfaction with Assistive Technology [51]; and the model of technology acceptance, specifically developed to test the acceptance of assistive social agents by older adults [73]. There is a lack of tools to evaluate the usability of assistive technologies in persons with severe dementia.

To conclude, despite advances in the field of technology-based interventions for persons living with dementia, few applications have been analyzed for their usability. Technologies can be used by many persons with dementia, but additional support is often needed from informal caregivers or professionals. To promote better utilization of technologies in dementia care, a better understanding is needed of their usability for persons with dementia, people’s preferences for specific interfaces, and their acceptance of different technologies.

### Effectiveness and Cost-Effectiveness of Assistive and Health Care Technologies in Dementia

**Figure 2:**
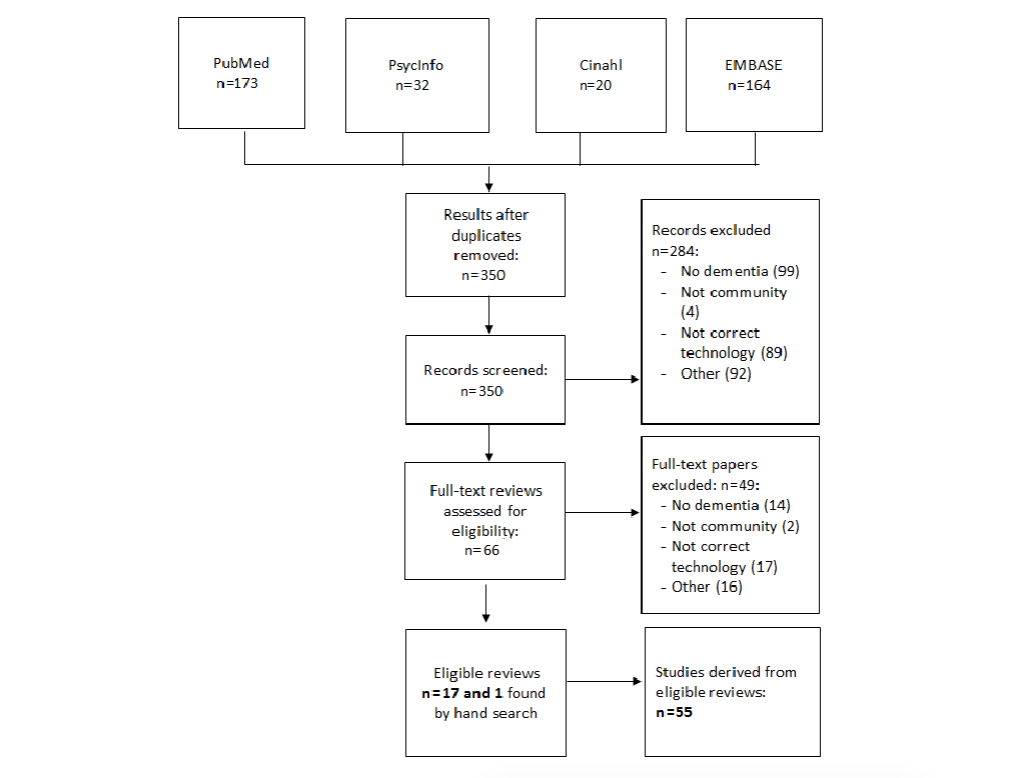
Flowchart of systematic review on effectiveness and cost-effectiveness.

The flowchart in Figure 2 illustrates the literature retrieved on effectiveness and cost-effectiveness of assistive and health care technologies. Eighteen reviews met our inclusion criteria, most of which (n=10) described a combination of the 3 technology domains we focused on in this study. One review focused on technologies to support persons with dementia in everyday life, 3 on technologies for engagement in pleasant and meaningful activities, and 4 on health care technology to support organizational and supportive systems. From the selected reviews, 55 individual studies described the effects of technologies on persons with dementia, the results of which are described in the following sections. None of the empirical studies described the cost-effectiveness of assistive and health care technologies for community-dwelling persons with dementia.

#### Assistive Technologies to Support Persons With Dementia in Everyday Life

Within this domain, many devices have been tested for their effectiveness. For example, a calendar was positively evaluated by more than half of the 21 participants [74]; a training device (based on errorless learning) to guide people with dementia in using a mobile phone was reportedly effective [75]; prompting devices to support in activities of daily life or in memory were found useful [76,77] and effective [76-80]; and prompts were found effective for traveling [81-84]. However, another prompting device found no impact on quality of life [85], which might have resulted from the many technological problems encountered during the effect pilot study. The NeuroPage [86] was tested in a randomized controlled trial (RCT) and showed a significant reduction in memory and planning failures by providing prompts; however, this study included patients with brain injury, and only a small number had dementia. Although tracking devices are said to be effective [87,88], 1 study showed that only a minority used such devices successfully, and 1 patient was injured by a passing vehicle [89]. Two studies also identified positive effects of tracking devices for caregivers (relief or reduction of emotional distress) [87,90].

#### Assistive Technologies to Support People With Dementia in Meaningful and Pleasurable Activities

Within the domain of technologies for meaningful or pleasurable activities, computer programs with cognitive training applications showed improvements in task performance or cognition in persons with Alzheimer’s dementia [70,91], recall [92], global cognitive functioning, and emotion [93,94]. However, devices with prompts for creative activities were found to be not effective [95,96], although participants liked the activities with an ePAD (Engaging Platform for Art Development) [95]. Social robot therapy for stimulating interaction showed an improvement in brain activity in half of the 14 participants [97]. Research into the use of multimedia tools to support people with dementia has reported improvements in well-being [98,99], mood [100], psychological stability [101], and social interaction and engagement [100,102-107]. In another study, a music tool was enjoyed by its users, but the prompts proved difficult to understand for the person with dementia [96]. Telephones or videophones have been reported as being easy to use for persons with dementia and helpful for maintaining social contacts, and they positively affected self-esteem [108-110].

#### Health Care Technologies

Health care technologies to facilitate health care delivery for people with dementia included sensors to monitor behavior, virtual reality, and video conferences. Sensors and smart home technologies are said to provide a good image of performed activities [111] and were reportedly successful in preventing major incidents [48,112,113]. Reported effectiveness of these tools in helping persons with dementia to live longer in the community varied [114,115]. One large controlled study [15] concluded that smart home technologies helped persons with dementia by improving confidence, ability to maintain community living for a longer time, and reducing need for care visits. A single case study found a reduction in required support, perceived anxiety, and confusion by the person with dementia [116]. Comparison of the use of video conferences for, for example, clinical assessment showed no differences compared with face-to-face assessments [117-119]. Clinical improvements were found for almost half of the study sample that received telecare, which consisted of telemedicine, tele-education, and telecounseling services [120], and this kind of telecare could be promising for rural populations [119].

#### Conclusion of Effectiveness and Cost-Effectiveness Assistive and Health Technology in Dementia

To summarize, various benefits of assistive technologies for persons with dementia have been reported. However, the results described need to be interpreted with caution because the majority of the included studies were uncontrolled studies with relatively small sample sizes. Reviews on cost-effectiveness studies of assistive and health care technologies in dementia were not found.

### Deployment of Assistive and Health Technology

Results regarding deployment were based on (1) recommendations for deployment of health technology identified by an expert panel and (2) a literature search using databases regarding health technology assessments (HTAs) and health services or technology assessments (HSTAs). These databases were chosen because they are specifically designed to give evidence-based recommendations and are directed at a nonscientific audience, for example, stakeholders who want to deploy health technology. The search resulted in 17 papers, of which 5 were relevant for the issues under consideration.

#### Deployment Issues

According to the Ambient Assisted Living Association (AALA) [121], “the market is growing beyond its traditional boundaries and this is attracting a growing interest by potential investors, the ICT industry and all service and care providers.” The landscape of the market will be deeply modified by a combination of a demand pull (by the rapidly growing population of older persons) and a technology push (through development of new ICT solutions and services) ([121], p. 76). A key recommendation of the AALA was to develop a European observatory with the mission to become the main source of trusted and high-quality information and data on the market to inform all stakeholders.

The next 3 paragraphs consider factors that influence deployment related to demand pull of stakeholders in general, health care professionals, and persons living with dementia.

#### Deployment Factors: Stakeholders in General

Stakeholders need trusted and high-quality information from HTAs or HSTAs. However, reviewing the current situation of HTA or HSTA delivers disappointing results in that these data, mainly provided by national bodies, are often incomplete, with many variations in definitions, information provided, and quality and reliability of the data [121].

The users of these data include health care providers, health service researchers, policy makers, funders, consumers, and information professionals (eg, in United States [122]; United Kingdom [123]; Germany [124]). Solely searching the HTA databases that provide English literature with the search term “assistive technology dementia” reveals few results (ie, United States: 14 books; United Kingdom: 3 items). Two of them provide facilitators and barriers (expanded upon later) to the deployment of technology: Jimison et al [125] and Finkelstein et al [126]. One is a systematic review on the effectiveness of assistive technology which does identify some of the barriers that are also mentioned in Jimison et al [125] and Finkelstein et al [126], and the other is a bibliographic record of an ongoing health technology assessment being undertaken [127]. One result was a Cochrane protocol focusing on the efficacy of assistive technology for memory support in dementia [128]. The other results were either not related to dementia or were not focusing on assistive technology.

#### Deployment Factors: Health Care Professionals

A range of constraints limiting deployment and related to the technology and health care sectors were identified at a workshop (2014) involving Ambient Assistive Living (AAL) and Joint Programme for Neurodegenerative Diseases (JPND) stakeholders; 25% of the projects funded by AAL and JPND are about developing ICT-based solutions for support and care of older adults with cognitive impairments [129]. These constraints came from a range of sectors including health and social care and business, covering aspects such as open standards, finance and business models, skills, and simply knowing what is available and where there are gaps in the market.

When assistive technology is used to enable support and care processes, barriers include the following: lack of usability; problems with access to the health IT application, low computer literacy in patients and clinicians, insufficient basic formal training in health IT applications; physicians’ concerns about more work; workflow issues; problems related to new system deployment, including concerns about confidentiality of patient information; depersonalization; incompatibility with current health care practices; lack of standardization; and problems with reimbursement [121]. Facilitators for the utilization of health IT included ease of use, perceived usefulness, efficiency of use, availability of support, comfort in use, and site location [126].

#### Deployment Factors: Persons Living With Dementia

Barriers for deployment of assistive technologies for the end user, which might also apply to a wider audience than dementia, include the following: usability problems, unreliable technology, the lack of consumers’ perceived benefit from using the system, inconvenience, data entry being cumbersome, and the intervention not fitting into the user’s daily routine. Deployment appeared to be more successful if the intervention could be delivered by technology that consumers already use daily for other purposes, and that satisfactorily meet their needs [125].

In conclusion, to promote successful deployment of assistive and health technologies in dementia care, it is essential that the technologies are reliable, user friendly, and useful; and that there is a single centrally funded access point to high-quality information regarding assistive technology products relating to dementia for all stakeholders. The Assistive Technology Dementia website [130] provides such a platform but is reliant on short-term funding (donations and grants), which means that optimization of information and sustainability are compromised. Furthermore, education and training in the field of technologies in dementia care should be available for all stakeholders.

### Ethical Considerations

**Figure 3:**
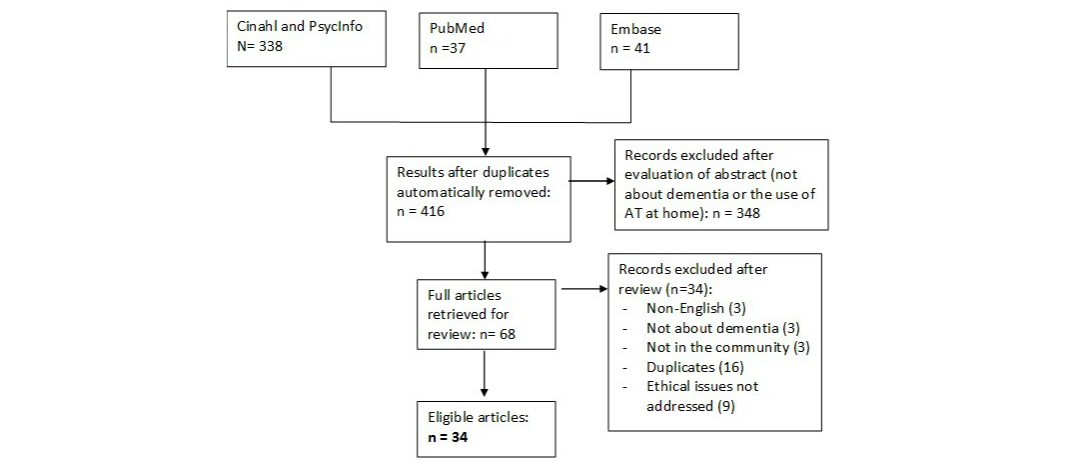
Flow chart of systematic review on ethics.

The analyses of the literature search regarding ethical considerations resulted in 33 references in which ethical issues were discussed linked to the use of assistive technology by or for persons with dementia living at home (see flowchart in Figure 3). The documents reviewed all covered at least one of the 3 assistive technology domains in the following numbers: technologies to support people in managing everyday life (13), to support in pleasurable and meaningful activities (1), health care technologies (1), and a combination of domains 1 and 2 (5), domains 1 and 3 (1), and all 3 domains (12). There was variation in the terminology used to cover ethical issues in relationship to assistive technology and in the coverage and the depth of consideration of such issues. Table 1 shows 7 categories of ethical issues resulting from the analysis and the reference numbers of the articles or studies in which they were addressed.

**Table 1:** Ethical issues addressed in the articles reviewed.

Category of ethical issue	Additional topics included	Articles or studies that addressed these ethical issues
Autonomy, freedom // paternalism, disempowerment	Informed consent, independence, the right to take risks, individuality, self-esteem and identity versus the use of restraint and coercive measures, over-protection	Cash [131]; Kang et al [132]; Landau et al [133]; Landau et al [134]; Landau et al [135]; Landau and Werner [136]; Lindqvist et al [137]; Mahoney et al [138]; Mao et al [139]; Martin and Cunningham [140]; McCabe and Innes [141]; McKinstry and Sheikh [142]; Mehrabian et al [143]; Miskelly [88]; Olsson et al [144]; Pino et al [145]; Plastow [146]; Pot et al [47]; Rauhala and Topo [12]; Robinson et al [14]; Robinson et al [147]; Robinson et al [148]; Siotia and Simpson [149]; Sorell and Draper [150]; Van Berlo [151]; Welsh et al [152]; Werner and Landau [134]; White and Montgomery [153]; Zwijsen et al [154]
Dignity, personhood // stigma, discrimination	Devaluation	Hughes et al [155]; Kang et al [132]; Landau et al [135]; Landau and Werner [136]; Mahoney et al [138]; Mao et al [139]; Marshall [156]; McCabe and Innes [141]; Plastow [146]; Robinson et al [14]; Robinson et al [147]; Robinson et al [148]; Sorell and Draper [150]; Werner and Landau [157]; White and Montgomery [153]; Zwijsen et al [154]
Social inclusion // replacement or loss of human contact	Simulated presence, staffing issues, and deception	Cash [131]; Kang et al [132]; Landau [133]; Landau et al [135]; Mahoney et al [138]; Marshall [156]; Martin and Cunningham [140]; Pino et al [145]; Plastow [146]; Robinson et al [147]; Siotia and Simpson [149]; Van Berlo [151]; Welsh et al [152]; Werner and Landau [157]; Zwijsen et al [154]
Privacy and data security	Confidentiality	Frisardi and Imbimbo [158]; Kearns and Fozard [159]; Landau et al [133]; Landau et al [134]; Landau et al [135]; Landau and Werner [136]; Mahoney et al [138]; McCabe and Innes [141]; McKinstry and Sheikh [142]; Mehrabian et al [143]; Rauhala and Topo [12]; Sorell and Draper [150]; White and Montgomery [153]; Zwijsen et al [154]
Overreliance on technology, new risks, false security		Landau et al [135]; Mao et al [139]; Marshall [156]; Martin and Cunningham [140]
Beneficence // nonmaleficence	Wellbeing, minimizing distress and harm (not only for people with dementia), for whose benefit the AT is used	Cash [131]; Hughes et al [155]) Kang et al [132]; Landau et al [133]; Landau et al [135]; Landau and Werner [136]; Lindqvist et al [137]; Mahoney et al [138]; Marshall [156]; McCabe and Innes [141]; Mehrabian et al [143]; Pino et al [145]; Pot et al [47]; Robinson et al [147]; Robinson et al [148]; Siotia and Simpson [149]; Sorell and Draper [150]
Equity or justice	Issues related to the individual and society (including costs)	Cash [131]; Mahoney et al [138]; Martin and Cunningham [140]; Mehrabian et al [143]; Rauhala and Topo [12]; Siotia and Simpson [149]; Van Berlo [151]; Welsh et al [152]; Werner and Landau [157]; Zwijsen et al [154]

A wide range of ethical issues were addressed but with a focus primarily on 3 of the 4 biomedical ethical principles (respect for autonomy, beneficence, and nonmaleficence) as well as on issues associated with care ethics and human rights (eg, social inclusion, human contact, personhood, dignity, and discrimination). Most researchers addressed a comprehensive range of ethical issues in the introduction to their article (ie, to contextualize their study or argument), but some gave much less attention to them when reporting their findings.

Several researchers (eg, Hughes et al [155]; Landau et al [133,135]; and Pino et al [145]) demonstrated a nuanced understanding of various ethical issues associated with the use of assistive technologies specifically for or by persons with dementia. This involved, for example, reflection on opposing concepts and concerns, such as social inclusion versus loss of human contact, or respect for autonomy versus concerns about safety (touching on coercion and paternalism). Some authors (McCabe and Innes [141]; Robinson et al [14]) emphasized that ethical issues are related to the way assistive technologies are used rather than inherent in particular devices or systems (eg, a device is not inherently stigmatizing; tracking devices may, depending on the situation and the individual, be experienced as either promoting or reducing freedom and autonomy).

Issues were frequently described in terms of ethical dilemmas of which 2 are notable. The first is about privacy and respect for autonomy versus safety and minimizing risks. The more safety a person with dementia wishes to have, the more it may be necessary for them (or others on their behalf) to accept some loss of privacy or autonomy and with various possible negative consequences (eg, safety at the expense of reduced quality of life, some risk but possibility to delay entry into residential care, deterioration of carer’s quality of life or health). The second ethical dilemma is about obtaining informed consent from persons with dementia due to possible difficulties understanding complex technology and loss of awareness over time of the presence or purpose of assistive technology, or that data is being collected on them.

## Discussion

### Principal Findings

The aim of our study was to describe the state of the art regarding development issues, usability, effectiveness and cost-effectiveness, deployment, and ethics of (assistive) technologies for community-dwelling persons with dementia, and based on that, to recommend a roadmap for development, research, and practice to support and promote the use of assistive technology, thus preparing society for the growing number of people with dementia.

A literature review was performed in the fields of usability, effectiveness and cost-effectiveness, and ethics. Most reviews were found in the field of usability, with the majority of these papers evaluating technologies to support daily living. In the field of effectiveness and cost-effectiveness, most reviews described a combination of the 3 technology domains we focused on in this study, and in the field of ethics, topics were addressed that were less related to the domain of technology, but rather to the way technology was used and the consequences for the user regarding, for example, autonomy and dignity.

Based on the results of the literature reviews and expert opinions, the following can be concluded about the state of the art of assistive technology for persons with dementia:

Development issues: Research has revealed that people with dementia are enthusiastic about using assistive technology to remain independent and also about taking part in technology design [23,33]. It is envisaged that the involvement of end users in the development of new assistive technologies will continue to grow, and that more applications of existing technology, using, for example, mobile phones and apps, will be put to use to benefit persons with dementia. We also anticipate that more companies will show an interest in this market, thus promoting the daily use of assistive technologies in dementia care. However there are also challenges such as how to personalize and tailor technologies to the individual needs and abilities of the person with dementia, how to address the emotional state of persons with dementia during everyday tasks [41], and how to integrate technology into the built environment and routine health care.

Usability issues: Little research so far has been conducted in community dementia care and support, with only a few studies exploring the usability of assistive technology in supporting everyday life [37,47,48]. The results showed that people with dementia were able to use the technology, but that additional support by informal caregivers or professionals was often needed. Furthermore, research showed that successful use of technology was related to computer literacy [65], and level of education of the users [50]. In the field of meaningful and pleasurable technology-based interventions, such as cognitive interventions for people with dementia, usability is generally not mentioned. However, a recent review showed promising findings for these activities using touchscreen technologies [160]. More research on usability in all areas of assistive technology is needed.

Effectiveness and cost-effectiveness: Various benefits of assistive technologies for people with dementia have been reported, such as cognitive and social functioning, mood and well-being, and reduction in service use. However, these findings need to be interpreted with caution because the majority of the included studies were uncontrolled, with half of them having included less than 10 persons with dementia. Most of the controlled studies included between 10 and 30 participants, and there were only 2 RCTs (1 with 46 and 1 with 143 participants of which less than 8% were people with dementia). No studies were found on the cost-effectiveness of assistive technologies or health technology interventions.

Deployment: Many barriers were identified ranging from a lack of knowledge about technology solutions, lack of usability and training, low computer literacy to incompatibility with current health care practices and reimbursement issues. Future projects should therefore focus more on the deployment of assistive technology, and appropriate business plans and scenarios need to be developed for bringing these technologies to the market. Looking to the future of the implementation of assistive technology in general, Peterson et al [161] concluded that future assistive technologies would be more integrated into the environment, combined with ambient and intelligent technologies, the potential of cloud computing, and the Internet of Things (a global network of physical objects that contain embedded technology to communicate and sense or interact with their internal states or the external environment). Assistive technologies will also become more personalized to individual needs and user requirements. These developments, however, will bring new challenges (see below).

Ethical issues: Many ethical issues were addressed by authors in the introduction of their papers, but less were described in the description of the results. With regard to assistive technologies in dementia, several authors stressed that ethical issues were not in the first place related to the technologies themselves but rather to how people use them. Ethical issues that were often described in this field are the dilemmas between autonomy and risk versus privacy reduction and increased safety and difficulties obtaining informed consent when persons with dementia do not understand or are not aware of the presence of the technology.

### The Identified Challenges

We identified several challenges for research into the selected research topics in the next few years.

Challenges in the development of assistive technology include how to develop these technologies in a way that meets individual variations in needs and abilities of persons with dementia, so that they really help to maintain autonomy, provide meaningful activities, and promote social inclusion. Another challenge is how to develop assistive technologies that address the emotional state of persons with dementia during everyday tasks [41]. A challenge in the field of health care technology supporting organizational systems and services in dementia care is to integrate the technology into the built environment, such as lighting, floor coverings, and improved way-finding [42,43], and into the routine health care, for example, by using ICT in the clinical assessment of cognitive, behavioral, and physical functioning of persons with dementia [44].

A challenge regarding usability lies in identifying those applications that have particular relevance for people living with dementia. A reiterated theme out of each of the literature reviews is the essential requirement to involve those with a diagnosis of dementia in identifying which needs technologies should meet, and in the development and usability testing of technology that is intended for people with dementia.

A challenge in effectiveness and cost-effectiveness research is to conduct methodologically sound scientific research in this field comparing assistive technology with care as usual. To conduct RCTs with large enough samples may be difficult because the assistive technologies may be expensive or it may be invasive to have them implemented in one’s home, for example, with sensors and cameras installed. Another challenge is to select adequate outcome measures that reflect the results of assistive technology interaction [161]. A third challenge is rooted in the fact that technology is an ever-moving target [20]. Everyday devices are continually developing with newer technologies coming to market, rendering evaluation of any one device obsolete within a short time frame. There is a clear need for new methods of rapid technology appraisal and evaluation to inform deployment [162].

Regarding deployment, the challenge lies in overcoming the barriers that will be faced as a result of the expected further integration of technologies within the built environment. These are challenges concerning, for example, data storage, system integrity, privacy and security, networked architecture, and service provision. Furthermore, having a good source of trusted and high-quality information on assistive and health care technologies to inform relevant stakeholders who may further implement them will be another challenge.

As for ethical issues, a challenge will be obtaining informed consent of participants with dementia for research on assistive technologies. This may have to do with difficulties in understanding what the technologies encompass and a lack of awareness over time of the presence and use of technology, or with data that are collected on people with dementia. Another challenge is to ensure that ethical issues are considered an important topic for researchers to include in their evaluation of assistive technologies.

### Limitations

The interpretation of assistive technologies used for the evidence reviews embraced bespoke devices developed to support persons living with dementia to manage their everyday life and participate in meaningful and enjoyable activities and health care technology. However, these reviews can only provide a retrospective snapshot of what has been researched rather than reflecting the current picture and what the future might hold. Also, the literature reviews were limited to (systematic) reviews rather than single studies because we aimed to get a global overview of the state of art. Furthermore, we did not consult persons living with dementia regarding their experiences and priorities.

### Recommendations

Our work underscored the challenge of determining the current “state of the art” in technology development and deployment given the dynamic definitions and various understandings of what assistive technologies are. This complexity is magnified when assistive technologies are situated within dementia. Nevertheless, based on the current literature, we recommend the following actions for development, usability, effectiveness and cost-effectiveness research, deployment, and ethics of assistive and health technologies across Europe and suggest that they are included in national and international calls for funding and assistive technology research programs in the coming decade (Textboxes 1-4).

**Actions to improve the development and usability of assistive technologies.**

- Persons with mild-to-moderate dementia or their supporters must be involved in all projects that aim to develop or test technologies for their ultimate benefit; this must be a prerequisite for project funding.
- Researchers involved in such technology development for persons with dementia must have adequate knowledge of dementia and, if not, receive specific training and support to enable full and meaningful engagement with persons with dementia; this should also be a prerequisite for funding.
- Steps must be taken to ensure that unnecessary replication of technology development that is proven unhelpful or ineffective does not occur.

**Actions regarding research into the effectiveness of assistive technologies.**

Research into the effectiveness of assistive technologies should move beyond explorative studies and include more and larger RCTs.

The focus should be on how technological services succeed in addressing individual needs of persons with dementia, as the population is heterogeneous and many face comorbid conditions.

Many different outcome measures are used in effect studies, making it difficult to synthesize the results of individual studies. Consensus on the use of outcome measures in this field is recommended [163]. Also, other designs such as randomized block designs with sufficient power can be considered to study these effects.

Research is needed on the cost-effectiveness of assistive technologies.

New methods of technology evaluation are required so that the results can be rapidly obtained and translated into practice, such as logging use and electronic ecological momentary assessments.

**Actions regarding the deployment of assistive technologies.**

Persons living with dementia and those involved in providing treatment and support need clear information about what already exists, for whom, and in what situations (eg, via the websites of national Alzheimer associations). They also need examples of how everyday devices can be used effectively by persons with dementia to enable appropriate deployment.

The benefits of new forms of technologies for persons with dementia have to be considered before they are brought on the market or disseminated; examples include robots for care and companionship and ubiquitous computing in the home and in society.

**Actions regarding ethics in using assistive technologies.**

Our review has demonstrated 3 important issues of relevance to researchers in this domain that ask for the following action:

There should be greater consistency among researchers regarding the terms used to describe ethical issues. This will facilitate the comparison of findings and recommendations.

Guidelines on ethical issues related to assistive technology use by or for people with dementia are available [164,165]. However, they are not widely applied in research exploring the role of assistive technology for community-dwelling persons with dementia. Researchers working in this area are advised to review and engage with these guidelines that provide a structured approach to addressing ethical dilemmas in the context of dementia care [165] rather than simply highlighting such ethical dilemmas. This should ensure that not only the conduct of the research complies with ethical principles but that the future use of devices also promotes ethical practice.

Researchers should strive to ensure that emerging reflection and findings on the ethical use of assistive technologies reach the general public, persons with dementia, informal carers, and health care professionals, and that for this wider dissemination, terms and explanations are understandable and meaningful to these targeted groups.

### Conclusions

Although this study shows that further research into the development and evaluation of assistive technologies for persons with dementia is needed, it also shows that they are enthusiastic about using technologies to remain their independency, that assistive technologies can improve cognition, mood, and social functioning and decrease service use, and that the use of technology is expected to improve with the increase in computer literacy and level of education, which will be the case in future generations of older people. It is therefore recommended that policy makers, care insurers, and care providers together with technology enterprises and researchers prepare strategies for the deployment of affordable assistive technologies in different care settings, to ensure that future generations of persons with dementia can derive benefit from this.

## Abbreviations

AALA: Ambient Assisted Living Association
CeHRes: Centre for eHealth Research and Disease Management
GPS: Global Positioning System
HCD: human-centered design
HSTA: health services or technology assessment
HTA: health technology assessment
ICT: information and communication technology
RCT: randomized controlled trial
